# Supplementary material for: Splice-Junction-Based Mapping of Alternative Isoforms in the Human Proteome
Source: Cell Rep. Author manuscript; Available in PMC 2020 Jan 15. (PMC6961840; doi:10.1016/j.celrep.2019.11.026)
Supplement: 2 [file NIHMS1546469-supplement-2.zip › DF1/PXD006675/AtrialSeptum/AtrialSeptum_13_MYBPC3_AITQLLCETEGR.pdf]

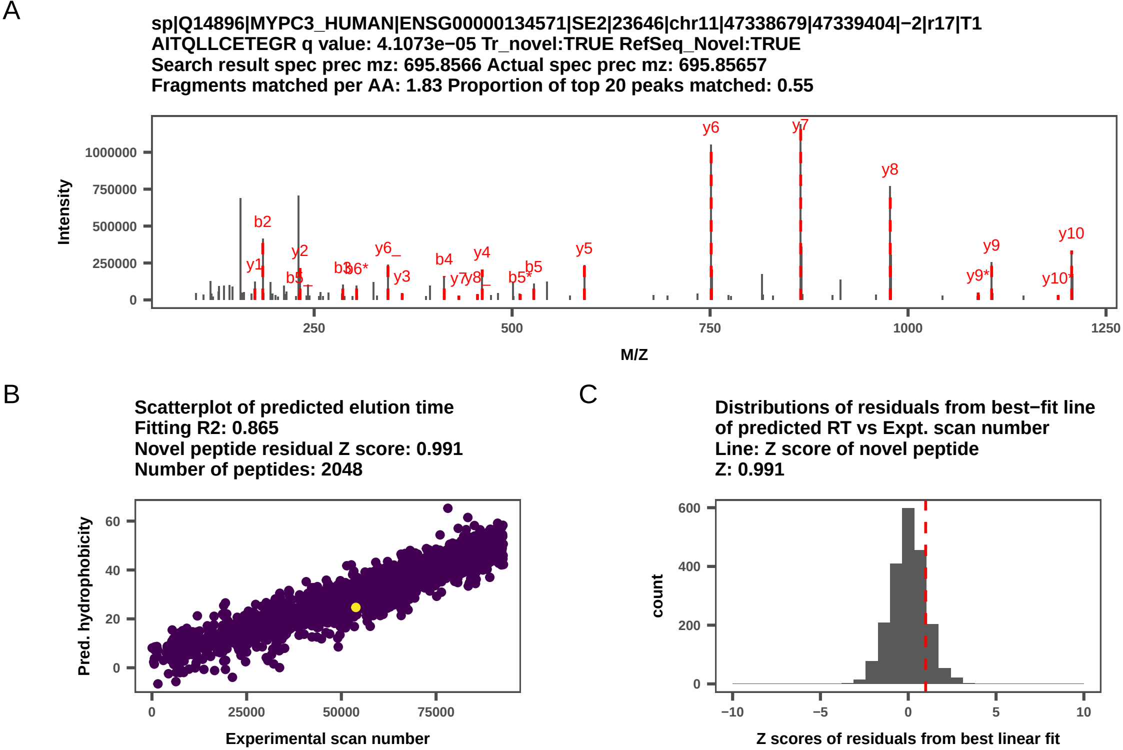
A
sp|Q14896|MYPC3_HUMAN|ENSG00000134571|SE2|23646|chr11|47338679|47339404|−2|r17|T1
AITQLLCETEGR q value: 4.1073e−05 Tr_novel:TRUE RefSeq_Novel:TRUE
Search result spec prec mz: 695.8566 Actual spec prec mz: 695.85657
Fragments matched per AA: 1.83 Proportion of top 20 peaks matched: 0.55
Intensity
1000000
750000
500000
250000
0
250
500
750
1000
1250
M/Z
y1
b2
y2
b5_
b3
b6*
y6_
y3
b4
y7
y8_
y4
b5*
b5
y5
y6
y7
y8
y9*
y9
y10*
y10
B
Scatterplot of predicted elution time
Fitting R2: 0.865
Novel peptide residual Z score: 0.991
Number of peptides: 2048
Pred. hydrophobicity
60
40
20
0
0
25000
50000
75000
Experimental scan number
C
Distributions of residuals from best−fit line
of predicted RT vs Expt. scan number
Line: Z score of novel peptide
Z: 0.991
count
600
400
200
0
−10
−5
0
5
10
Z scores of residuals from best linear fit
